# Supplementary material for: Sensory experience during early sensitive periods shapes cross-modal temporal biases
Source: eLife. 2020 Aug 25;9:e61238. doi: 10.7554/eLife.61238 (PMC7476755; doi:10.7554/eLife.61238)
Supplement: Supplementary file 1. [file elife-61238-supp1.docx]

Supplementary File 1. Hierarchical logistic models – predictor evaluation.

|  | Bias | | Resolution | |
| --- | --- | --- | --- | --- |
|  | visual-auditory  Experiment 1 | visual-tactile  Experiment 2 | visual-auditory  Experiment 1 | visual-tactile  Experiment 2 |
| Group | χ^2^(3) = 13.33,  *p* = 0.004 | χ^2^(3) = 20.88,  *p* < 0.001 | χ^2^(3) = 18.74,  *p* < 0.001 | χ^2^(3) = 19.61,  *p* < 0.001 |
| Modality Condition |  |  | χ^2^(2) = 33.75,  *p* < 0.001 | χ^2^(2) = 34.80,  *p* < 0.001 |
| Group x Modality Condition |  |  | χ^2^(4) = 26.06,  *p* < 0.001 | χ^2^(6) = 11.12,  *p* = 0.085 |

To test for temporal order biases toward one modality, we conducted a hierarchical logistic regression on single trial ‘visual first’-values with group as predictor. To analyze spatio-temporal resolution across groups and modality conditions, we conducted a hierarchical logistic regression on single trial accuracy values using group and modality as predictors. The significance of the predictors was evaluated using likelihood ratio tests.
